# Defining the evolving field of culinary medicine across multiple domains

**DOI:** 10.3389/fnut.2025.1588449

**Published:** 2025-09-30

**Authors:** Caitlin A. Hildebrand, Kristi E. Artz, Beth Dollinger, Kerri Dotson, David D. Dungan, Sabrina A. Falquier, Basma Faris, Nurgul Fitzgerald, Karl J. Guggenmos, Gregory A. Harlan, Timothy S. Harlan, Karen E. Joseph, Barbara Kamp, Karen L. Lindsay, Amy Moyer, Vibha Patel, Gloria Richard-Davis, Michelle Troup, Jessica R. VanRoo, Ileana Vargas, Jaclyn L. Albin

**Affiliations:** ^1^Cecil G. Sheps Center for Health Services Research, University of North Carolina, Chapel Hill, NC, United States; ^2^Department of Nutrition, Gillings School of Global Public Health at the University of North Carolina, Chapel Hill, NC, United States; ^3^University of North Carolina Center for Health Promotion and Disease Prevention, Chapel Hill, NC, United States; ^4^University Hospitals Connor Whole Health, Cleveland, OH, United States; ^5^Arnot Health, Horseheads, NY, United States; ^6^Lake Erie College of Osteopathic Medicine, Elmira, NY, United States; ^7^American College of Culinary Medicine, McLean, VA, United States; ^8^Duly Health and Care, Lombard, IL, United States; ^9^Sensations Salud, LLC, San Diego, CA, United States; ^10^Herbert Wertheim School of Public Health and Human Longevity Science, University of California, San Diego, San Diego, CA, United States; ^11^Department of Integrative Wellness, Point Loma Nazarene University, San Diego, CA, United States; ^12^Department of Nutrition, Bastyr University, San Diego, CA, United States; ^13^Department of Obstetrics, Gynecology and Reproductive Science, Icahn School of Medicine at Mount Sinai, New York, NY, United States; ^14^Department of Nutritional Sciences, Rutgers University, New Brunswick, NJ, United States; ^15^Johnson & Wales University, Providence, RI, United States; ^16^World Association of Chefs Societies, Paris, France; ^17^Department of Medical Education, Keck School of Medicine, University of Southern California, Los Angeles, CA, United States; ^18^Department of Pediatrics, Keck School of Medicine, University of Southern California, Los Angeles, CA, United States; ^19^School of Medicine and Health Sciences, George Washington University, Washington, DC, United States; ^20^Beautifully Fed, LLC, New York, NY, United States; ^21^Department of Pediatrics, School of Medicine, University of California, Irvine, Irvine, CA, United States; ^22^UCI Susan Samueli Integrative Health Institute, University of California, Irvine, Irvine, CA, United States; ^23^Department of Nutrition, University of North Carolina at Greensboro, Greensboro, NC, United States; ^24^Knownwell Health, Plano, TX, United States; ^25^Morehouse School of Medicine, Atlanta, GA, United States; ^26^School of Medicine Greenville, University of South Carolina, Greenville, SC, United States; ^27^Naomi Berrie Diabetes Center, Columbia University Irving Medical Center, New York, NY, United States; ^28^Department of Pediatrics, University of Texas Southwestern Medical Center, School of Medicine, Dallas, TX, United States; ^29^Department of Internal Medicine, University of Texas Southwestern Medical Center, School of Medicine, Dallas, TX, United States; ^30^O'Donnell School of Public Health, University of Texas Southwestern Medical Center, Dallas, TX, United States

**Keywords:** culinary medicine, simulation-based medical education with deliberate practice (SBME-DP), experiential learning, interprofessional collaboration, nutrition education, interdisciplinary training, interprofessional education (IPE)

## Abstract

Culinary medicine is an evolving field that spans multiple professions and settings. Prior definitions of culinary medicine may not reflect the expansion of this discipline or the variety of key stakeholders engaged in culinary medicine. To address the need for an updated definition of what culinary medicine means, a working group consisting of a variety of roles and professional/community settings was assembled to reach a consensus definition that is reflective of the current landscape of culinary medicine. Definitions that are tailored to the variety of stakeholders participating in culinary medicine are beneficial to engagement within and across professions in support of programming, clinical care, and access to healthy food with the ultimate goal of empowering patients/consumers to make healthier food choices.

## 1 Introduction

Culinary medicine has often been defined as “a new evidence-based field in medicine that blends the art of food and cooking with the science of medicine” (1). However, with documentation of culinary medicine experiences in the literature for over two decades and expansive implementation of the practice in multiple contexts and engaging a variety of stakeholders, culinary medicine is no longer new, and the field has grown and evolved considerably, warranting the need to revisit the definition of this discipline.

Recent scoping reviews of culinary medicine experiences for medical trainees demonstrate the growing popularity of and demand for culinary medicine as well as the variety of stakeholders participating in culinary medicine experiences (2–4). One review documented that the first article describing a culinary medicine experience for medical trainees was published in 2002, yet the majority of publications were published in 2018 or later, reflecting the growth and momentum seen in this field in recent years (4). The reviews showcase the variety of stakeholders with demonstrated interest in culinary medicine, including healthcare professionals and trainees, foodservice professionals, patients, and community members (2–4).

The implementation approaches for culinary medicine experiences vary. Culinary medicine typically involves incorporating food preparation skills with nutrition education, such as a combination of hands-on cooking and a case-based learning experience. The instructor and the learner in culinary medicine can look very different depending on the context and intent of the experience, such as healthcare professional educators and foodservice professionals partnering to train future practitioners to counsel patients on diet or culinary and healthcare professionals teaming up to lead community classes for patients.

An updated definition of culinary medicine is beneficial for a variety of stakeholders, from those interested in introducing culinary medicine for the first time to longstanding programs seeking to continue meeting the needs of their participants. A consensus definition of this evolving concept may enhance advocacy for programming in terms of engaging support, securing resources, and fostering collaborations.

Evidence supporting the efficacy of culinary medicine in a variety of contexts is growing (2, 4, 5). The need for continued evaluation of programming to examine the long-term impact and sustainability of culinary medicine would benefit from a consensus definition of what culinary medicine means.

To address the need for an updated definition of the expanding and multidisciplinary field of culinary medicine, this working group sought to reach a consensus definition of culinary medicine that reflects the variety of stakeholders and contexts of this evolving field. We report the definitions reached and describe the key stakeholders and domains identified. The working group's iterative approach to reaching a consensus definition through dialogue amongst multidisciplinary professionals and community members adds to the literature an updated definition of what culinary medicine means, reflective of a variety of perspectives.

## 2 Process

### 2.1 Working group

An expert panel of individuals involved in culinary medicine programming was assembled with the goal of arriving at a definition to provide a unified understanding of this evolving discipline. Recognizing the diversity of experiences and backgrounds of stakeholders involved in culinary medicine, the working group took an inclusive approach to membership to capture a variety of perspectives. The group also sought to reduce bias by inviting members representative of a variety of backgrounds, roles, and professional/community contexts.

The final working group consisted of 30 individuals involved in culinary medicine programming and research across multiple roles, including dietitians, physicians, chefs, foodservice professionals, nutrition researchers, nutrition educators, community members, and a health professional student (Table 1).

**Table 1:** Working group member roles and primary focus of work.

**Working group member roles**	
Physician	11
Dietitian	8
Chef/foodservice professional	6
Medical student	1
Community member	4
Nutrition researcher	7
**Work group members primary focus of work**	
Academic—healthcare professions	16
Private practice healthcare	4
Foodservice—institutional	1
Foodservice—other	3
Academic—culinary profession	3
Community member	2
Culinary nutrition educator	1

Working group member roles also crossed over in their focus of work and professional or community settings, such as working in academia, private practice, institutional foodservice settings (e.g., schools, hospitals), other foodservice (e.g., restaurants, academia), or community-facing roles like extension service or other non-healthcare professionals (Table 1). As an example of crossover, the working group included members trained as chefs and health care professionals with experience teaching in community and academic settings.

### 2.2 Approach

Over the course of 8 months, the working group met six times. Initial meeting discussions included the impact of culinary medicine and the need for a workable definition of the term. Before discussing how to define culinary medicine, a literature review was conducted by 2 working group members and a student to better understand how culinary medicine has been previously defined. One working group member and a student independently searched “culinary medicine,” and the two working group members reviewed relevant articles for whether and how culinary medicine was defined. To reflect more recent trends in the growing field of culinary medicine, the literature search was limited to contemporary articles and reports published between January 2016 and December 2023. The phrase “culinary medicine” was found in 44 articles indexed in PubMed. The literature review confirmed that most reports referenced the La Puma definition of culinary medicine: “culinary medicine is a new evidence-based field in medicine that blends the art of food and cooking with the science of medicine” (1). This commonly used definition was presented to the working group to consider in conceptualizing updates needed given the growth in stakeholders adopting culinary medicine across multiple disciplines.

The initial working group meeting was a moderated discussion of current and past definitions for culinary medicine, as identified through the literature review, with the goal of creating a framework on which the committee could build upon to reach an inclusive definition. A working group member who is an academic physician and chef volunteered to serve as moderator. Meetings and communications included discussions ranging from the meaning of culinary medicine to the variety of populations involved in teaching or learning within the discipline, such as healthcare professionals, foodservice professionals, culinary nutrition educators, and community members.

The working group further refined definitions and concepts through follow-up meetings and formal email communications before reaching a consensus on defining culinary medicine in the context of current applications in the literature and through expert experience. Meetings served as opportunities for open dialogue in the multidisciplinary group to inform definitions proposed in written, email-based communications. Email communications were used for the formal voting process and as an additional space for feedback on definitions. The voting process by email communications was iterative, consisting of two sequential votes on multiple versions of each of the stakeholder definitions, and with each vote working group members could also provide feedback to consider for revisions. Members received a third email with the final definitions and had the opportunity to suggest minor revisions. This process was used as a means of open dialogue among a multidisciplinary group actively engaged in culinary medicine efforts to voice opinions and reach consensus on the definitions reported below (Table 2).

**Table 2:** Consensus definitions of culinary medicine by key stakeholder population.

**Key stakeholder population**	**Definition**
To the healthcare professional:	Culinary medicine is a multifaceted field that bridges the gap between the most up-to-date, evidence-based nutrition science and culinary tradition. It is based on food is medicine principles: the augmentation of nutrition in health care to maintain health and to prevent and treat disease.
To the foodservice professional:	Culinary medicine is the convergence of fields, the breaking down of silos, and the uniting of the “two white coats.” Culinary medicine is the cost-conscious, smart solution to good health based on nutrition science and classical culinary techniques.
To the culinary nutrition educator:	Culinary medicine is the application of science and traditional knowledge coming together with familiar, generational cooking to inspire communities to achieve and sustain nutritious eating behaviors and better health.
To the consumer/community member:	Culinary medicine is empowering individuals to make decisions about nourishing their body to prevent chronic disease and maintain good health while still feeding their soul.

## 3 Outcomes

### 3.1 Definitions

While initially aiming for a single definition of culinary medicine, the working group determined through the process that multiple definitions, tailored to the various stakeholders in culinary medicine, were needed to capture the variety of perspectives in this multidisciplinary field. The working group reached consensus to define the dynamic term of culinary medicine through the lenses of four key stakeholder groups identified: healthcare professionals, foodservice professionals, culinary nutrition educators, and community members. Consensus definitions of culinary medicine by stakeholder group are listed in Table 2.

### 3.2 Key stakeholders and domains

The working group considered not only the key stakeholders engaged in culinary medicine (Table 2) but also the key domains in which culinary medicine activities primarily take place, which the working group identified as: academic, clinical, and community. The following sections describe examples of applications of culinary medicine by different stakeholders in the domains considered by the working group.

The academic setting typically encompasses healthcare professional training and foodservice training. For example, in healthcare professional training, simulation-based medical education with deliberate practice (SBME-DP) is a well-documented methodology of teaching culinary medicine that is most predominantly used in medical education but can also be applied to other healthcare professional training (6, 7). SBME-DP connects didactic learning and hands-on cooking classes with clinical case studies, reinforcing the lessons learned in a way that traditional pedagogical teaching does not. Instructors initially guide healthcare professional trainees in the kitchen through simple demonstrations, but the bulk of the culinary learning occurs through experiential hands-on cooking activities. Likewise, the trainees work on team-based case studies that are then presented as the deliberate practice activity with guidance from instructors. Academic healthcare professional trainees vary in profession (e.g., dental, dietetics, nursing, physicians, physician assistants, pharmacy, etc.) and level of training, spanning from undergraduate pre-professional settings to continuing education for those in practice (4, 5, 8). Culinary medicine training can also be delivered as interprofessional education, bringing together multidisciplinary instructors and trainees including inviting the other “white coat” from the culinary profession (3, 4, 9), with the added benefit that interprofessional training in culinary medicine is supporting health professional trainees in attaining competencies in not only nutrition but also interprofessional collaboration (10–12).

Culinary medicine engagement in the academic foodservice setting can entail foodservice faculty as instructors in academic healthcare professional training or foodservice trainees learning about health and nutrition as it relates to preparing healthful foods. In culinary training, nutrition education is a required curricular component for programs certified by the American Culinary Federation (13). Culinary medicine training offers the potential to engage culinary trainees in learning nutrition through practical application.

Supplementing traditional didactic teaching with hands-on cooking experiences using health condition-related challenges allows aspiring culinarians to bring together nutrition science and their kitchen skills to create meals that satisfy the health condition needs without sacrificing food appeal. This active learning of nutrition reinforces the lessons learned in a way that is not achieved with conventional teaching methods.

Beyond the academic setting, foodservice professionals span multiple domains, playing a critical role in the health of our population. Chefs and those in foodservice are feeding consumers at multiple levels, such as institutional foodservice, chain and fast-food outlets, chef-owned restaurants, and consumer products companies. Now, one of their many “toques” includes not only feeding consumers but also providing culinary training to healthcare professionals and patients/community members through culinary medicine (4, 14, 15). Engaging foodservice professionals in culinary medicine in meaningful ways can have additional applications in the clinical environment beyond teaching healthcare professionals and patients, such as managing hospital-based foodservices and helping develop food as medicine programs. Finally, for the foodservice profession, culinary medicine training could transform menus and food offerings to be healthier for consumers.

In the clinical environment, culinary medicine interventions can be delivered during patient encounters through established processes, including counseling and shared medical decision-making. Culinary medicine is increasingly being offered through shared medical appointments (SMAs). SMAs delivering hands-on cooking classes in culinary medicine kitchens or virtually allow for expansion of reach and potential reimbursement for services (16, 17).

Current barriers to addressing nutrition in healthcare, such as time constraints, knowledge gaps, reimbursement concerns, and limitations in access to registered dietitian nutritionists (RDNs), may present challenges to integrating culinary medicine in traditional patient visits (18–20). However, innovative approaches are emerging to make culinary medicine more accessible and reimbursable. One method is physician-to-physician electronic consults (eConsults) with certified culinary medicine specialist (CCMS) physicians or culinary medicine trained physician-RDN teams (18, 21). This option allows for flexibility, tailored patient plans, and reimbursement for services with minimal resource requirements (18). Physician-to-patient electronic communications are another flexible, low-resource, and potentially reimbursable option for physicians, or physician-RDN teams, to provide patients with tailored guidance using the electronic medical record patient portal system. While more resource-intensive, consults can also be achieved through more traditional in-person or virtual visits. More intensive nutritional counseling can be achieved through RDN referrals. Telehealth visits with RDNs, which increased in availability for patients during the COVID-19 pandemic, may present opportunities for improved interprofessional collaboration in nutrition care and greater accessibility to the support, services, and nutrition expertise RDNs provide (22). Culinary medicine principles could be introduced by the referring clinician or integrated in the RDN visit, whether in person or virtual. Pharmacists, physician assistants, nurses, and other health professional disciplines with culinary medicine training can enhance team-based nutrition care and expand opportunities for dietary counseling, and health professional students from multiple disciplines are already participating in interprofessional culinary medicine learning experiences as previously described.

In the community, culinary medicine has multiple reaches. Culinary nutrition educators deliver culinary medicine education via didactic presentations coupled with hands-on cooking classes (23–25). Community programming can look very different depending on the organization, setting, and populations served. Community culinary nutrition programs can also provide a referral option for healthcare professionals to provide patients with the opportunity to build practical skills to make dietary changes. In the end, the consumer is the ultimate constituency reached by culinary medicine, whether as restaurant patrons, food shoppers, or patients aiming to nourish themselves and loved ones with foods they enjoy.

## 4 Discussion

Culinary medicine is a growing field that is inclusive of a variety of facilitators, learners, and settings. As such, defining the field must consider these varying contexts. The results of this working group's process of defining culinary medicine consider the perspectives of the key stakeholders identified in implementing culinary medicine: healthcare professionals, foodservice professionals, culinary nutrition educators, and consumers/community members.

### 4.1. Benefits of defining culinary medicine

Defining culinary medicine through the lens of each of the key stakeholders has key benefits.

#### 4.1.1 Healthcare

In the healthcare professions, a clear definition of what culinary medicine means can help inform the design and implementation of programming in hospitals, clinics, and training programs when engaging administrators and faculty to gain institutional support. A healthcare professional-tailored definition can also improve clinician understanding of how culinary medicine can help them better meet the nutrition needs of their patients. Focusing on evidence-based nutrition science is essential for a group for whom evidence is a cornerstone of practice.

Bridging nutrition science principles with culinary tradition is especially important as it takes into account the need for healthcare professionals to consider both the health and sociocultural impacts of food when discussing diet with patients. Engaging clinicians in culinary medicine can support them in delivering practical nutrition guidance through the hands-on learning of nutrition that culinary medicine offers. Better equipping clinicians to empower patients to improve their health through food could also help nutrition gain greater attention in medical practice, as many non-dietitian clinicians cite barriers to addressing nutrition such as deficits in knowledge and confidence (19, 26). Culinary medicine holds promise as a way to strengthen collaborations across multiple healthcare professional disciplines (e.g., physicians and dietitians) and invites less traditional interprofessional collaborations between healthcare and foodservice.

#### 4.1.2 Foodservice

Foodservice professionals have an important role in culinary medicine by offering expertise in culinary skills and creativity in preparing healthful and appetizing foods. Defining culinary medicine for the foodservice profession can help realize this potential through an improved understanding of how foodservice professionals can meaningfully contribute to health. Foodservice professionals are an important resource for healthcare professionals, culinary nutrition educators, and community members to gain culinary knowledge and skills in healthy and delicious food preparation.

A clear understanding of the meaning of culinary medicine in foodservice also supports relationship-building by bringing together health, nutrition, and culinary expertise, as seen in the teams of physicians, dietitians, and chefs delivering culinary medicine training to medical trainees (27–29). Finally, recognizing foodservice professionals' role in culinary medicine can open doors to more healthful food offerings for consumers, as training foodservice professionals in culinary medicine tenets could have the effect of making it easier for consumers to follow a healthier dietary pattern with more healthful options that also deliver on taste.

#### 4.1.3 Culinary nutrition educators

Engaging culinary nutrition educators in culinary medicine opens opportunities to expand the reach of nutrition education through hands-on cooking in tailored and meaningful ways. Culinary nutrition is a related term that may be more familiar to some stakeholders depending on their professional roles and domains (30). Given the growing popularity of the term culinary medicine, consideration of the culinary nutrition educator's perspective of what culinary medicine means is essential to improving collaboration across disciplines and domains. An understanding of the definition of culinary medicine for this stakeholder population can also support the introduction and sustainability of programming in multiple settings, through harnessing existing relationships built on shared knowledge, experience, and backgrounds. Given the sociocultural significance of food, a deeper understanding of a community's needs and preferences can empower communities to make healthier food choices without sacrificing familiar tastes and food preparations. Culinary nutrition educators can help overcome challenges otherwise faced by healthcare professionals who may lack knowledge of food traditions of a given population. They can also serve as a referral resource for healthcare professionals to support patients in gaining culinary knowledge and skills to follow nutrition guidance outside of busy and time-limited patient encounters.

#### 4.1.4 Community

Ultimately, each of these stakeholder populations—healthcare professionals, foodservice professionals, and culinary nutrition educators—serve to reach individual consumers in the promotion of health through food. Understanding what culinary medicine means for the consumer, particularly that healthy food can be familiar and enjoyable, can support meaningful engagement between patients and their healthcare professional teams, consumers and foodservice professionals, and community members and nutrition educators. Defining culinary medicine for patients, consumers, and community members is critical to the success of programs; therefore, it is paramount that we can clearly communicate the definition of culinary medicine programming and the positive impact it can have for this group. Culinary medicine not only can improve consumer knowledge of nutrition to make healthier choices but also can empower individuals with the skills to prepare health-promoting foods they love.

### 4.2 Using culinary medicine definitions to support implementation

While evidence supporting the benefits of culinary medicine across multiple domains continues to grow, barriers to program implementation present challenges to widespread uptake. Access to institutional support, facilitators/instructors, funding, resources, and kitchen space are common barriers (3, 4, 9). An understanding of what culinary medicine means, tailored to a domain's audience, can help facilitate dialogue to advocate for programming. Culinary medicine definitions tailored to key stakeholders can also encourage collaboration between “silos,” helping fulfill various needs. For example, foodservice institutions can provide content expertise or kitchen space. Collaborations between stakeholders, such as pooling resources between healthcare professional training programs or teaching kitchens or partnering with foodservice institutions or community organizations, are documented facilitators of culinary medicine programming (3, 4).

Within stakeholder groups, a clear understanding of culinary medicine can support conversations about initiating, sustaining, or evaluating programming. For instance, many medical trainees are advocating for culinary medicine experiences to gain practical nutrition counseling skills but need administrative approval, curricular integration, and funding (4). Similarly, community organizations operating culinary medicine nutrition education programs often rely on support through grants and philanthropy, necessitating a clear understanding of what culinary medicine means in this context. Improved understanding of the scope of culinary medicine could improve program uptake, participation, and measurement of impact. While many programs have demonstrated positive impacts (2, 4, 5, 31, 32), more research is needed to understand the long-term impact of culinary medicine across domains.

Finally, culinary medicine definitions reflective of diverse stakeholders and domains can support advocacy for policy-level changes in support of culinary medicine implementation. Improved understanding of culinary medicine and the ability to articulate its benefits across multiple domains could support engagement with health care systems, health insurance payers, and lawmakers to advocate for increased access to programming in clinical, educational, and community contexts.

### 4.3 Limitations and challenges

While we were intentional in selecting working group members to reflect an interdisciplinary perspective, some stakeholder groups were more represented than others due to their high engagement and interest in the need to advance clarity in the field. In support of the interdisciplinary focus, many working group members crossed over in their roles and domains, representing multiple perspectives while also reflecting in-depth experience in how many domains interact due to dedication to interdisciplinary practice and broad practice experiences. Some members' roles evolved over time with historical perspectives of other domains distinct from their current roles, also adding richness to perspectives. Given the fluidity and crossover among working group members, clearly designating each role was challenging.

Defining culinary medicine presents challenges given the rapid evolution of the field, as evidenced by continued growth in culinary medicine-focused publications. Since the conclusion of the working group meetings, there were 15 relevant manuscripts published in 2024. Articles cover topics such as virtual programming (33), international programming (34), impact on adoption of Mediterranean diet principles (35), and definitions of culinary nutrition terms in practice and education (30).

We anticipate that the field will continue to evolve at an increasingly rapid pace. The evolution of the concept of culinary medicine will need to be reassessed on a regular basis to keep up with increased implementation of programs as well as the expanding domains using culinary medicine programming. We plan to monitor for relevant innovations, including new terms that emerge. Moreover, building culinary medicine knowledge requires an iterative approach for all learners in all domains, and we hope the growing stakeholders in the field continue efforts to define applications of culinary medicine rooted in the mission of transforming human health through delicious, nourishing food.

## 5 Conclusion

As the discipline of culinary medicine continues to establish itself across multiple settings and contexts, an improved understanding of the meaning of culinary medicine is essential. The definition must also reflect the various perspectives of groups actively engaged as implementers and consumers of culinary medicine. As such, this working group, intentionally diverse in background and experience, reached a consensus in defining what culinary medicine means in ways identifiable to the various stakeholders engaged, inviting all to the table.

## Data Availability

The original contributions presented in the study are included in the article/supplementary material, further inquiries can be directed to the corresponding author.
